# cGAS/STING Signaling in Ulcerative Colitis: Mechanism and Therapeutic Opportunities

**DOI:** 10.3390/ijms27146513

**Published:** 2026-07-22

**Authors:** Xinyi Dai, Jiaqi Zhang, Xudong Tang

**Affiliations:** Institute of Digestive Diseases, Xiyuan Hospital of China Academy of Chinese Medical Sciences, Beijing 100080, China; sl410710125@163.com

**Keywords:** ulcerative colitis, cGAS, STING, innate immunity, natural products

## Abstract

Ulcerative colitis (UC) is a chronic, relapsing inflammatory bowel disease characterized by mucosal inflammation and epithelial barrier disruption. The cyclic GMP-AMP synthase (cGAS)-stimulator of interferon genes (STING) pathway, the principal cytosolic DNA-sensing axis, has emerged as a critical node in UC pathogenesis. This review elucidates the cGAS/STING signaling network in UC, from its upstream activation triggered by exogenous stimuli and leaked endogenous DNA to its downstream effects. We discuss how this pathway modulates distinct lines of intestinal defense, including the mechanical, chemical, and immunological barriers, while integrating its bidirectional crosstalk with the microbial barrier. Rather than exerting purely detrimental effects, cGAS/STING functions as a double-edged sword that coordinates both barrier homeostasis and inflammatory pathogenesis. Crucially, we summarize current pharmacological interventions, highlighting synthetic small molecules and bioactive natural products that target and modulate this axis to restore intestinal equilibrium. This framework provides a theoretical foundation for future precision therapies in UC.

## 1. Introduction

Ulcerative colitis (UC) is a chronic, nonspecific inflammatory bowel disease that primarily affects the mucosa and submucosa of the colon and rectum. Clinically, UC is characterized by recurrent episodes of abdominal pain, diarrhea, bloody stools containing mucus or pus, tenesmus, and weight loss. Epidemiological evidence indicates that the global burden of UC continues to increase, with approximately five million individuals affected worldwide in 2023, thereby placing a substantial burden on patients’ quality of life [1]. Although the precise etiology of UC remains incompletely understood, disease onset and progression are generally considered to arise from a complex interplay among genetic susceptibility, dysregulated mucosal immunity, environmental exposures, and intestinal microbial dysbiosis [2]. Conventional therapies, including 5-aminosalicylic acid (5-ASA), corticosteroids, and immunosuppressants, remain the cornerstone of UC treatment, while biologics and small-molecule targeted agents have substantially expanded the therapeutic landscape in recent years [3,4,5]. Nevertheless, limited therapeutic durability, primary or secondary treatment failure, and systemic adverse effects remain major clinical challenges [1]. Therefore, identifying novel therapeutic targets involved in intestinal inflammation is essential for improving therapeutic outcomes.

Dysregulated mucosal immunity is widely recognized as a central driver of UC pathogenesis. As the first line of host defense, the innate immune system relies on pattern-recognition receptors (PRRs) to detect pathogen-associated molecular patterns (PAMPs) and damage-associated molecular patterns (DAMPs), thereby initiating immune responses to microbial invasion and sterile tissue injury within the intestinal mucosa [6,7]. Under physiological conditions, these responses are tightly controlled and contribute to host defense and tissue repair. In UC, however, persistent activation of innate immune signaling promotes feed-forward inflammatory cascades, disrupts epithelial barrier integrity, and perpetuates mucosal injury. Among PRR-mediated signaling networks, the cyclic GMP-AMP synthase (cGAS)-stimulator of interferon genes (STING) pathway has emerged as a pivotal cytosolic DNA-sensing mechanism. Beyond its role in host defense against infection, aberrant activation of this pathway has been increasingly implicated in tumor immune evasion, sterile autoimmunity, and a broad spectrum of chronic inflammatory disorders [8,9,10]. Upon sensing cytosolic DNA, cGAS synthesizes cyclic GMP-AMP (cGAMP), which activates STING and subsequently triggers TBK1/IRF3 and NF-κB signaling, leading to type I interferon and inflammatory cytokine production [11]. Accumulating evidence indicates aberrant activation of the cGAS/STING pathway in UC, supporting an important role for this pathway [9,12]. The context-dependent roles of cGAS/STING signaling present both challenges and opportunities for therapeutic intervention. Targeting this pathway has therefore attracted increasing attention, with synthetic inhibitors and natural products showing therapeutic potential in experimental colitis models [13]. In this review, we summarize current evidence on the role of the cGAS/STING pathway in UC pathogenesis and discuss recent advances in synthetic small-molecule inhibitors and natural product-based modulators, highlighting their therapeutic potential and translational challenges for UC treatment.

To provide a structured overview of current evidence, relevant literature was identified by searching PubMed, Web of Science, and Scopus using combinations of the keywords “cGAS”, “STING”, “colitis”, “inflammatory bowel disease”, “intestinal inflammation”, “inhibitor”, “small molecule”, “herbal”, “Chinese medicine” and “natural product”. English-language publications available up to May 2026 were considered. We focused on original research involving human samples, animal models, organoids, and cultured cells, as well as relevant reviews addressing the mechanistic and therapeutic roles of cGAS/STING signaling in UC. Given the heterogeneity of the available experimental evidence and the rapidly evolving nature of the field, a structured narrative approach was adopted rather than a formal systematic review methodology.

## 2. Overview of the cGAS/STING Pathway

Components of the cGAS/STING signaling pathway are broadly expressed across immune, non-immune, and malignant cell types. The canonical function of this pathway is to sense cytosolic DNA and initiate innate immune responses through the induction of type I interferons (IFN-I) and interferon-stimulated genes (ISGs). Beyond this classical role, accumulating evidence indicates that the cGAS/STING axis also regulates diverse cellular processes, including autophagy, pyroptosis, cellular metabolism, and senescence [14,15].

As a cytosolic dsDNA sensor, cGAS recognizes various endogenous and exogenous DNA species, including microbial DNA, mtDNA, damaged chromosomal DNA, RNA-DNA hybrids, and extrachromosomal circular DNA [16,17]. Structurally, cGAS contains multiple DNA-binding interfaces, including the canonical A-site and the auxiliary B- and C-sites. These interfaces cooperate during cGAS activation rather than functioning independently. The coordinated engagement of the A-, B-, and C-sites facilitates cGAS dimerization, higher-order assembly, and efficient enzymatic activation. Specifically, DNA binding at the A-site promotes conformational activation of the catalytic domain, whereas the B- and C-sites provide additional DNA contacts that stabilize cGAS-DNA assemblies and enhance signal amplification. Thus, productive cGAS activation requires the cooperative engagement of multiple DNA-binding interfaces rather than engagement of a single site alone [18,19]. This cooperative mechanism also explains the DNA-length dependence of cGAS activation, as longer dsDNA more effectively promotes the clustering and alignment of cGAS molecules into functional signaling assemblies [20]. Once activated, cGAS catalyzes the synthesis of the second messenger 2′3′-cGAMP from ATP and GTP, which subsequently acts as the endogenous ligand for STING [21,22]. Although cGAS was initially characterized as a cytosolic DNA sensor, recent studies have identified a substantial nuclear pool of cGAS. Under physiological conditions, nuclear cGAS is restrained through interactions with nucleosomes, which limit its access to genomic DNA and prevent inappropriate oligomerization, thereby maintaining immune homeostasis and preventing autoreactivity against self-DNA [23].

STING (also known as TMEM173) is an approximately 40-kDa homodimeric transmembrane adaptor protein localized to the endoplasmic reticulum (ER) membrane. Structurally, STING consists of a transmembrane domain (TMD) and a carboxy-terminal domain (CTD), which contains the ligand-binding domain (LBD) and a flexible C-terminal tail (CTT) [24,25]. Binding of cGAMP to the LBD induces a conformational rearrangement of STING and promotes its trafficking from the ER to the Golgi apparatus, where it recruits and activates downstream signaling molecules [14,26]. During this process, the exposed CTT recruits and activates TBK1, resulting in the phosphorylation and activation of IRF3 [27,28]. In parallel, activated STING oligomers promote IKK complex activation and subsequent NF-κB nuclear translocation [29]. Activated NF-κB and IRF3 cooperatively regulate the transcription of genes encoding IFN-I and pro-inflammatory cytokines, including tumor necrosis factor-alpha (TNF-α), IL-1β, and IL-6. Subsequently, secreted type I interferons bind to the IFN-α/β receptor (IFNAR) to induce the expression of interferon-stimulated genes (ISGs) [29]. Importantly, the modular molecular architecture of STING enables differential regulation of its downstream signaling pathways. Although the CTT has been considered the primary platform for downstream effector recruitment, emerging evidence indicates that STING-mediated NF-κB activation is not absolutely dependent on its CTT. Instead, distinct regulatory elements enable both CTT-dependent and CTT-independent signaling mechanisms, allowing context-specific regulation of inflammatory and interferon responses.

**Figure 1:**
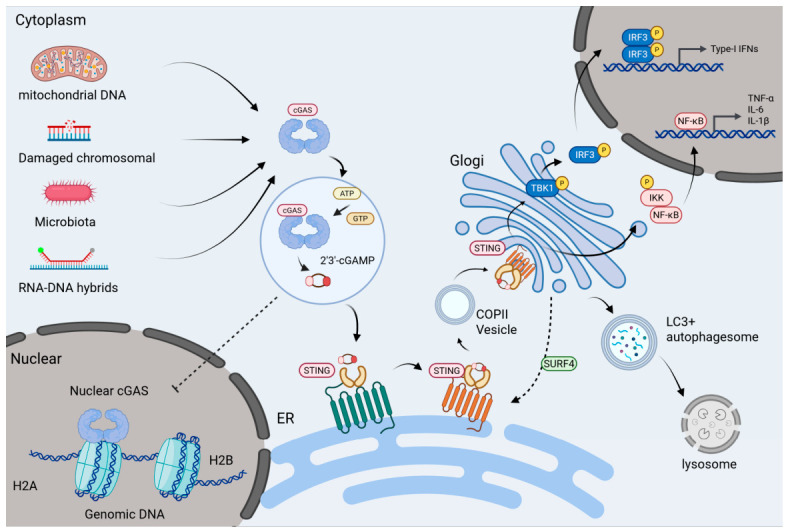
Overview of the cGAS/STING signaling. Cytosolic dsDNA fragments arise from either foreign extracellular sources or endogenous DNA released during cell stress and death. They cooperatively bind to cGAS, driving its dimerization and liquid-like condensation to synthesize 2′3′-cGAMP. Subsequently, cGAMP binds homodimeric STING on the ER membrane, triggering higher-order oligomerization. This conformational change exposes COPII motifs, facilitating STING trafficking via the ERGIC to the Golgi apparatus. At the Golgi, STING recruits and activates TBK1 to phosphorylate IRF3, driving its nuclear translocation to initiate IFN-I and ISG transcription. Concurrently, STING activates the IKK complex to induce NF-κB-mediated pro-inflammatory cytokine expression. Additionally, nuclear cGAS is sequestered by H2A–H2B nucleosomes to restrict inappropriate autoimmune responses. STING translocation from the ER triggers a non-canonical LC3^+^ autophagosome-lysosome degradation pathway that degrades active STING to terminate inflammatory signaling. 2′3′-cGAMP, 2′3′-cyclic GMP-AMP; cGAS, cyclic GMP-AMP synthase; COPII, coat protein complex II; dsDNA, double-stranded DNA; ER, endoplasmic reticulum; ERGIC, endoplasmic reticulum-Golgi intermediate compartment; H2A–H2B, histone H2A–H2B; IFN-I, type I interferon; IKK, IκB kinase; IRF3, interferon regulatory factor 3; ISG, interferon-stimulated gene; LC3^+^, microtubule-associated protein 1 light chain 3-positive; NF-κB, nuclear factor-kappa B; STING, stimulator of interferon genes; TBK1, TANK-binding kinase 1.

To prevent excessive inflammation, STING activation is tightly controlled by intracellular trafficking and feedback mechanisms. STING trafficking from the ER to the Golgi not only mediates immune activation but also induces noncanonical autophagy characterized by the formation of LC3-positive vesicles [14]. Under steady-state conditions, SURF4-mediated retrieval of Golgi-localized STING through COPI vesicles contributes to maintaining immune homeostasis [30]. Following sustained activation, STING is ultimately transported to lysosomes for degradation, thereby terminating inflammatory signaling (Figure 1). 

## 3. Upstream Stimuli Driving cGAS-STING Activation in UC

### 3.1. Microbial DNA and Cyclic Dinucleotides

Under physiological conditions, the intestinal mucosal barrier strictly segregates the luminal microbiota from host tissues. In UC, however, microbial dysbiosis and epithelial barrier disruption may allow microbial DNA and cyclic dinucleotides to access host sensing pathways. Once bacterial or viral genomic DNA gains access to the host–cell cytoplasm, it acts as a PAMP and activates cGAS, triggering the downstream TBK1-IRF3 cascade and inducing IFN-I production and ISG expression [31,32]. In parallel, microbial cyclic dinucleotides, such as cyclic di-GMP and cyclic di-AMP, can bypass cGAS and directly bind to and activate host STING protein, initiating downstream inflammatory cascade [33].

Microbial extracellular vesicles (mEVs), including outer membrane vesicles (OMVs) released by Gram-negative bacteria, provide an important route for delivering microbial DNA into host cells [34]. OMVs are nanoscale structures, generally 40–300 nm in diameter, that contain bacterial DNA, periplasmic proteins, lipopolysaccharides, and outer membrane components [35]. These vesicles can deliver microbial DNA to intestinal epithelial cells or tissue-resident innate immune cells, thereby facilitating cGAS activation. An increased abundance of mEVs has been detected in the colonic mucosa of patients with IBD and mice with experimental colitis. Under homeostatic conditions, tissue-resident CRIg^+^ macrophages contribute to the clearance of these immunogenic vesicles. In UC, however, depletion of CRIg^+^ macrophages permits mEV accumulation and sustained cGAS-STING activation, whereas pharmacological inhibition of this pathway or restoration of CRIg expression attenuates mucosal inflammation [36]. During enteric viral infections, cGAMP generated in infected cells may also be incorporated into viral particles or host-derived extracellular vesicles and transferred to adjacent cells. This process activates STING without requiring the entry of additional exogenous DNA, thereby propagating the inflammatory cascade [37].

### 3.2. Endogenous DNA

#### 3.2.1. Genomic DNA

Chronic intestinal inflammation and oxidative stress increase DNA double-strand breaks (DSBs) and compromise genomic integrity [38]. During cell division, unresolved DNA damage can lead to micronucleus formation. Rupture of the micronuclear envelope exposes genomic DNA to cGAS, while DNA fragments generated by genomic damage may also accumulate in the cytosol during interphase, thereby sustaining cGAS activation [17,39]. The maintenance of genomic stability is therefore important for preventing inappropriate cGAS activation. For instance, the RNA/DNA helicase DHX9, which is downregulated in patients with UC, contributes to DNA replication and transcriptional homeostasis by resolving RNA or DNA duplexes as well as DNA:RNA hybrids (R-loops) [40]. In the context of UC, DHX9 deficiency promotes R-loop accumulation, genomic instability, and DSB formation, thereby activating cGAS-STING-dependent sterile inflammation and impairing intestinal stem-cell function [41].

#### 3.2.2. Mitochondrial DNA

Compared with nuclear genomic DNA, mtDNA is more susceptible to oxidative damage and cytosolic release because of its limited histone protection, high copy number, and proximity to mitochondrial reactive oxygen species. Cytosolic mtDNA can therefore act as a potent endogenous DAMP [42]. Its release may occur during programmed cell death in intestinal epithelial cells, neutrophil extracellular trap formation, and dysregulated mitochondrial fission [43,44,45]. In postmenopausal female UC patients, reduced estrogen receptor β (ER-β) expression in the colonic mucosa has been associated with disrupted mitochondrial dynamics and increased mitochondrial fragmentation. This structural collapse precipitates massive leakage of mtDNA, sequentially hyperactivating the cytosolic cGAS/STING axis to accelerate intestinal epithelial senescence and barrier disintegration [46]. In addition, mitochondrial transcription factor A (TFAM)-bound mtDNA released from damaged colonic epithelium in extracellular vesicles or molecular complexes can be taken up by mucosal myeloid cells. This process activates STING and promotes downstream interleukin-12 (IL-12) signaling, thereby promoting pathogenic Th1 and Th17 cell differentiation [47].

Mechanistically, the translocation of matrix-enclosed mtDNA into the cytosol requires the sequential assembly of sophisticated macromolecular pores across the mitochondrial membranes. BAX/BAK macropores formed during apoptosis and oligomerized VDAC channels under sublethal mitochondrial stress can facilitate mtDNA passage across the outer mitochondrial membrane, whereas prolonged opening of the mitochondrial permeability transition pore may contribute to inner-membrane permeabilization [48,49,50,51]. Reactive oxygen species can also generate oxidized mtDNA (ox-mtDNA) fragments that are resistant to degradation, promoting the phosphorylation of STING at Ser365. This modification promotes the assembly of the cGAS-STING-IRF3 supramolecular complex and initiates the type I interferon cascade [52]. Of note, most of the aforementioned mechanisms of mtDNA release have been derived from non-intestinal cell models, and direct validation in the UC intestinal epithelium remains limited. In L929 cells, phosphorylated mixed-lineage kinase domain-like protein (MLKL) was shown to translocate to mitochondria and induce microtubule-dependent mtDNA release, leading to cGAS/STING activation. The disease relevance of this mechanism was further supported in an intestinal epithelial cell-specific Caspase-8-deficient mouse model of spontaneous IBD, in which MLKL-dependent necroptosis involved the mtDNA/cGAS/STING axis [43]. However, direct evidence demonstrating MLKL mitochondrial translocation and mtDNA release in intestinal epithelial cells is still lacking.

### 3.3. Other Exogenous Factors

Beyond microbial entities, xenobiotic exposures associated with contemporary dietary lifestyles have emerged as key factor triggers for cGAS-STING hyperactivation within the intestinal mucosa. For instance, in a DSS-induced murine colitis model, oxidized triglyceride monomers generated during the frying process, activate the cGAS-STING signaling pathway, leading to the production of pro-inflammatory cytokines and increased intestinal permeability [53]. Similarly, the dietary long-chain fatty acid palmitic acid (PA), abundant in Western-style diets, exerts a significant pro-inflammatory effect in the pathogenesis of IBD [54]. Studies have reported that PA treatment induces endothelial cell mitochondrial damage and mtDNA release, thereby activating the STING/IRF3 pathway [55]. However, direct validation of PA-induced mtDNA release and cGAS–STING activation specifically within intestinal epithelial cells remains lacking. Therefore, findings from endothelial cell models should be extrapolated to the intestinal epithelium with caution. Furthermore, perfluoroalkyl substances (PFAS), a ubiquitous class of anthropogenic environmental contaminants, are epidemiologically linked to IBD progression [56]. In particular, perfluorodecanoic acid (PFDA, a key representative of the PFAS family) has been reported to significantly exacerbate experimental intestinal inflammation through the aberrant activation of the cGAS/STING/NF-κB transcriptional axis both in vivo and in vitro [57].

## 4. The Mechanism of cGAS/STING Signaling in Ulcerative Colitis

### 4.1. Aberrant cGAS/STING Activation in UC Patients

Evidence from human colonic tissues indicates that the cGAS/STING pathway is dysregulated in UC. Compared with healthy controls, patients with UC exhibit significantly higher expression levels of both cGAS and STING in their colonic mucosa [58,59,60,61], with higher levels observed in active disease than during remission [62]. Consistent with these findings, phosphorylated STING (p-STING), a molecular marker of pathway activation, is also markedly increased in patients with active UC compared with those in remission [62]. Furthermore, mucosal STING expression positively correlates with IL-10 levels and shows increased colocalization with CD4^+^ T cells, as demonstrated by immunofluorescence staining [63]. Persistent pathological activation of the cytosolic DNA sensor cGAS may be driven by an overload of DAMPs. Elevated levels of circulating cf-DNA have been detected in the plasma of patients with UC [64,65], although another study found no significant correlation between cfDNA levels and disease activity [65]. As an important component of the circulating cfDNA pool, mtDNA is also significantly increased in patients with UC and positively correlates with histological disease activity scores [66]. In addition to excessive DNA release, impaired extracellular DNA clearance may further contribute to DNA accumulation. Specifically, the enzymatic activity of deoxyribonuclease I (DNase I), the primary endonuclease responsible for degrading extracellular DNA, is significantly reduced in the serum of patients with IBD [67]. Collectively, current clinical evidence supports increased cGAS/STING expression and activation in UC, particularly during active inflammation. Nevertheless, it remains unclear whether these changes represent a primary pathogenic event or a secondary response to mucosal injury. Moreover, the utility of circulating DNA levels or mucosal STING activation as biomarkers requires validation in larger cohorts, with appropriate consideration of disease extent, treatment exposure, and inflammatory activity.

### 4.2. cGAS/STING Signaling Regulates Intestinal Mechanical and Chemical Barriers

#### 4.2.1. Aberrant cGAS/STING Activation Exacerbates Intestinal Epithelial Barrier Damage

The intestinal mechanical barrier, primarily formed by intestinal epithelial cells and their intercellular tight junction complexes, constitutes the first line of defense against luminal pathogens and harmful antigens. In UC, aberrant activation of the cGAS/STING pathway disrupts this barrier by amplifying inflammatory signaling and inducing multiple forms of regulated cell death. Experimental colitis models have associated increased cGAS/STING expression with NF-κB activation and elevated production of pro-inflammatory cytokines, including TNF-α, IL-1β, and IL-6 [62,68]. Consistently, genetic deletion of *cGAS* or *STING* in murine colitis models significantly reduces NF-κB and TNF expression in colonic tissues [61,63]. Sustained cytokine production not only recruits immune cells but also directly impairs tight junction integrity and increases epithelial permeability.

Beyond inflammatory amplification, cGAS/STING signaling contributes to intestinal barrier disruption through the induction of multiple forms of regulated cell death. Infiltrating macrophages can activate epithelial cGAS/STING signaling through the release of macrophage extracellular traps (METs), resulting in reduced epithelial cell viability, enhanced apoptosis, and decreased expression of tight junction proteins in a dose- and time-dependent manner in DSS-induced colitis [69]. However, the precise molecular mechanisms underlying MET-induced apoptosis were not further elucidated in this study. Under conditions where apoptotic signaling is impaired, STING activation can promote RIPK3-dependent necroptosis through amplification of type I interferon and TNF signaling pathways [70]. In primary intestinal epithelial cells (IECs), mtDNA-mediated STING activation has also been shown to synergize with IFN-γ and TNF-α signaling to trigger necroptotic cell death [71]. Moreover, deficiency of the IBD susceptibility gene *ATG16L1* exacerbates endoplasmic reticulum stress, resulting in excessive STING activation and severe necroptosis, ultimately aggravating experimental colitis [12]. Crucially, the relationship between necroptosis and nucleic acid sensing is bidirectional; necroptotic cell lysis may in turn release endogenous DNA and reactivate cGAS, thereby sustaining an inflammatory feedback loop [43].

Concurrently, the cGAS/STING axis is closely associated with NLRP3 inflammasome assembly and subsequent gasdermin D (GSDMD)-mediated pyroptosis [72,73]. In murine colitis models and cultured intestinal epithelial cells, excessive STING activation induces NLRP3/Caspase-1/GSDMD signaling and aggravates epithelial injury [74]. Similar findings have been reported in organoid models derived from patients with UC, in which the pyroptosis-related proteins pro-caspase-1, GSDMD-N, and NLRP3 were markedly upregulated. Notably, pharmacological activation of STING reversed the protective effects conferred by NLRP3 inhibition, accompanied by restored expression of pyroptosis-associated proteins and increased intestinal permeability [75]. Consistent with these observations, brefeldin A (BFA), a protein transport inhibitor, alleviated DSS-induced colitis through suppression of cGAS signaling [60], an effect that may also be related to its reported inhibitory activity against the NLRP3 inflammasome and STING signaling [29,76]. Nevertheless, conflicting findings have also been reported. One study reported that in human intestinal organoid models, cell death and IL-1β/IL-18 release induced by IFN-β or IFN-γ in combination with TNF-α were dependent on JAK kinase activity but independent of NLRP3 inflammasome and caspase activation [58].

Ferroptosis has emerged as an iron-dependent form of regulated cell death characterized by excessive lipid peroxidation [77]. In DSS-induced colitis, the neuropeptide substance P alleviated intestinal inflammation by suppressing cGAS/STING signaling, reducing PTGS2 and ACSL4 expression while increasing SLC7A11 levels, thereby attenuating ferroptotic injury in intestinal epithelial cells [78].

Despite the above evidence supporting a pro-inflammatory and pro-death role of cGAS/STING in the intestinal epithelium, one study using TNF-α/CHX stimulation to induce apoptosis in wild-type and constitutively activated STING-mutant (N153S) intestinal organoids found comparable levels of lactate dehydrogenase (LDH) release between groups, suggesting that STING activation alone may be insufficient to directly induce epithelial cell death [79]. Differences in experimental stimuli, cellular context, and inflammatory conditions may account for these discrepancies.

#### 4.2.2. cGAS/STING Signaling Supports Intestinal Barrier Homeostasis

Despite its pathogenic effects under excessive activation, cGAS/STING signaling may also support epithelial homeostasis. *cGAS*-deficient mice exhibit more severe colitis, and mechanistic studies have shown that ectopic expression of cGAS in IECs increases LC3-II/I expression and promotes autophagy. Mechanistically, cGAS deficiency disrupts Beclin-1-dependent physiological autophagy, thereby increasing epithelial cell death [61]. Similarly, *STING^−/−^* mice display reduced goblet cell numbers, impaired mucus secretion, and decreased secretory IgA production [80]. Another study further demonstrated that STING deficiency reduced colonic crypt depth and downregulated the tight junction protein occludin, rendering mice more susceptible to DSS-induced mucosal injury [81]. This apparent paradox reflects the regulatory complexity of the cGAS/STING pathway. On the one hand, type I interferons may exert context-dependent effects, both synergizing with NF-κB to amplify inflammatory responses and promoting dendritic cell maturation, effector T-cell differentiation, and B-cell antibody production [82]. NF-κB activation itself also exhibits a dual role. While sustained or excessive activation drives inflammatory injury and tissue damage, complete inhibition of this pathway may disrupt immune homeostasis and impair mucosal repair processes [83].

Overall, the effects of cGAS/STING activation in UC depend on the balance between protective and pathogenic signaling. The relative contributions of the IFN-I and NF-κB pathways, as well as the cell type-specific and non-canonical functions of cGAS and STING in epithelial repair, remain incompletely understood. Physiological activation may support intestinal homeostasis, whereas sustained overactivation promotes inflammation and tissue injury.

### 4.3. cGAS/STING Signaling Mediates Immune Barrier Regulation

Following disruption of the intestinal mechanical and chemical barriers, luminal microorganisms and their products can more readily penetrate the mucosa and gain access to the lamina propria, thereby triggering local immune responses. Disruption of immune homeostasis within the intestinal microenvironment is a hallmark of UC, characterized by aberrant pro-inflammatory activation of innate immune cells, including macrophages and dendritic cells, together with maladaptive responses of the adaptive immune system such as an imbalance among T-cell subsets. The cGAS/STING signaling axis acts as a central regulator of immune cell recruitment and phenotypic reprogramming, thereby coordinating intestinal inflammatory responses. At the level of innate immunity, excessive macrophage activation is critically involved in the initiation and propagation of mucosal inflammation. Classically activated M1 macrophages promote tissue injury by releasing large amounts of pro-inflammatory mediators, whereas alternatively activated M2 macrophages facilitate the resolution of inflammation and tissue repair [84]. Accumulating evidence indicates that activation of the cGAS/STING pathway promotes macrophage infiltration and promotes macrophage M1 polarization. In experimental colitis models, STING-deficient mice exhibit markedly reduced expression of the macrophage activation marker Iba-1 in colonic tissues. Conversely, activation of STING with the murine STING agonist DMXAA shifts reparative M2 macrophages toward an M1-like phenotype, thereby exacerbating intestinal inflammation [85]. Mechanistically, this process may involve activation of the GM-CSF/STAT5 signaling axis downstream of cGAS/STING, which promotes M1 macrophage differentiation and pro-inflammatory cytokine production [85]. Interestingly, the host also possesses intrinsic regulatory mechanisms that counterbalance nucleic acid-driven macrophage activation. Developmental endothelial locus-1 (DEL-1), a multidomain glycoprotein secreted by tissue-resident cells has emerged as an important endogenous anti-inflammatory mediator. Functionally, macrophage-derived DEL-1 acts as a bridging molecule, in which its C-terminal domain recognizes phosphatidylserine exposed on apoptotic cells, while its N-terminal domain binds β3 integrins on macrophages, thereby facilitating efferocytosis and accelerating inflammation resolution [86,87]. Recent evidence from experimental colitis models further indicates that DEL-1 can selectively suppress activation of the CMPK2/cGAS/STING signaling axis, thereby promoting reparative macrophage polarization and ameliorating colitis severity [88]. Meanwhile, Gasdermin D (GSDMD), the key executor of pyroptosis in macrophages, has also been shown to negatively regulate upstream cGAS signaling. In experimental colitis models, GSDMD suppressed cGAS-mediated IFN production in macrophages by promoting K^+^ efflux and reducing dsDNA binding to cGAS, thereby attenuating cGAS/STING activation and exerting protective effects against colitis.

Beyond macrophages, dendritic cells are pivotal antigen-presenting cells that link innate and adaptive immunity and are therefore play an important role in shaping intestinal inflammatory responses. In the spontaneous STING-activating N153S mutant mouse model, one study indicated that constitutively activated STING preferentially accumulated within myeloid cells and promoted intestinal inflammation associated with enhanced colonic Th1-cell infiltration [79]. Similarly, another study reported that mtDNA released from colonic epithelial cells activates the STING/IRF3/NF-κB signaling cascade in DCs. Genetic deletion of STING in myeloid cells suppressed IL-12 family cytokine production by DCs, thereby modulating Th1 and Th17 differentiation and restoring colonic immune homeostasis [47].

**Figure 2:**
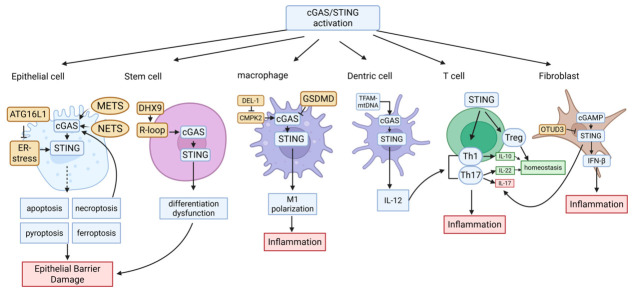
Cell-type-specific roles and downstream regulatory networks of the cGAS/STING pathway in UC. Hyperactivation of epithelial cGAS/STING triggers multiple modalities of programmed cell death (apoptosis, pyroptosis, necroptosis, and ferroptosis), which is extrinsically driven by METs and NETs. Notably, ATG16L1 deficiency exacerbates ER stress to amplify the STING cascade and execute epithelial necroptosis. The necrotic demise provokes pathological mtDNA release, sustaining a feed-forward inflammatory cycle. In ISCs, DHX9 depletion induces aberrant R-loop accumulation and genomic instability, activating cGAS/STING to impair self-renewal functionality. In macrophages, cGAS/STING signaling predominantly drives pro-inflammatory M1 polarization, which is counterregulated by GSDMD (restricting dsDNA-cGAS engagement) and the endogenous anti-inflammatory factor DEL-1 (via a CMPK2/cGAS/STING negative feedback axis). In DCs, epithelial-derived, TFAM-bound mtDNA complexes serve as DAMPs to engage cGAS/STING, prompting IL-12 secretion and subsequent pathogenic Th1/Th17 differentiation. In T cell subsets, STING activation promotes IL-10 secretion by CD4^+^ T cells and regulates Treg abundance, while non-canonical STING signaling modulates Th17 cells to secrete IL-22 for mucosal barrier integrity. In fibroblasts, OTUD3 deficiency amplifies the cGAMP-mediated cGAS/STING/IFN-β cascade to drive CD4^+^ T cell IL-17A secretion, mediating the inflammatory response. cGAMP, cyclic GMP-AMP; cGAS, cyclic GMP-AMP synthase; DAMPs, damage-associated molecular patterns; DCs, dendritic cells; dsDNA, double-stranded DNA; DEL-1, developmental endothelial locus-1; ER, endoplasmic reticulum; GSDMD, gasdermin D; IFN-β, interferon-beta; IL-10, interleukin-10; IL-12, interleukin-12; IL-17A, interleukin-17A; IL-22, interleukin-22; ISCs, intestinal stem cells; METs, macrophage extracellular traps; mtDNA, mitochondrial DNA; NETs, neutrophil extracellular traps; OTUD3, OTU domain-containing protein 3; STING, stimulator of interferon genes; TFAM, mitochondrial transcription factor A; Tregs, regulatory T cells; UC, ulcerative colitis.

In contrast to its predominantly pro-inflammatory role in innate immune cells, STING may exert anti-inflammatory functions in adaptive immunity. In DSS-induced colitis models involving either global STING deficiency or CD4^+^ T cell-specific STING deletion, intestinal inflammation was paradoxically aggravated. Mechanistically, STING activation was found to promote STAT3 translocation from the cytoplasm to both the nucleus and mitochondria, thereby enhancing Blimp-1 expression and mitochondrial oxidative metabolism, which together facilitated IL-10 production by Th1 cells [68]. Consistent with previous findings, another study found that STING signaling may interact with commensal microbiota to promote the production of anti-inflammatory cytokines such as IL-10. Combined deficiency of both IL-10 and STING did not result in more severe colitis than IL-10 deficiency alone [89], suggesting that STING may function primarily to counterbalance excessive inflammatory responses and preserve intestinal immune homeostasis. STING deficiency has also been reported to disrupt intraepithelial and lamina propria lymphocyte homeostasis, reducing the frequency and suppressive function of regulatory T (Treg) cells, although no significant changes in lamina propria Th-cell populations were observed [80]. However, aberrant STING activation in other cell types can communicate with T cells and amplify inflammatory cascades. For instance, pathological STING activation in fibroblasts may prompt CD4^+^ T cells to produce IL-17, thereby driving further inflammatory progression [90] (Figure 2).

These discrepancies are possibly driven by a sophisticated spatial-temporal heterogeneity, in which the downstream effects of STING are determined by both cell lineage and disease stage. For example, a paradigm of this temporal dualism is illustrated by the finding that myeloid STING deletion at different time points exerts diametrically opposed influences on the initiation versus the progression of colitis-associated cancer [47]. Future studies are warranted to further elucidate the cell-type-specific and stage-dependent dynamics of cGAS/STING signaling during the development and progression of UC.

### 4.4. Interactions Between cGAS/STING Signaling and the Microbial Barrier

The intestinal microbiota and its metabolites collectively constitute an important microbial barrier that contributes to the maintenance of intestinal homeostasis. Intestinal microbial dysbiosis is a hallmark of UC, and accumulating evidence indicates that the cGAS/STING pathway functions not only as a sensor of microbiota-derived signals but also as an active regulator of microbial composition and metabolic homeostasis. The structural features of the cGAS DNA-binding surface enable microbiota-derived DNA to engage and activate host cGAS; however, the gut microenvironment can also amplify STING-dependent inflammatory signaling through several distinct mechanisms. In the spontaneous STING-activating N153S mouse model, depletion of intestinal microbiota with broad-spectrum antibiotics significantly alleviated colitis, indicating that the gut microbiota contribute to STING-driven intestinal inflammation. Mechanistically, microbiota-derived metabolites may enhance K63-linked ubiquitination of STING in myeloid cells, thereby promoting STING stabilization and sustaining the downstream inflammatory signaling cascade [79]. Host genetic susceptibility further amplifies microbiota-mediated pathological STING activation. Mice carrying UC-associated Otud3 variants developed severe colonic lesions following fecal microbiota transplantation (FMT) from patients with UC. As a deubiquitinase, OTUD3 deficiency or functional impairment markedly increases fibroblast sensitivity to microbiota-derived cGAMP, resulting in excessive amplification of STING signaling [90]. Nevertheless, STING activation is not universally detrimental. Specific strains of *Lactobacillus* modulate IFN-I production through a STING-dependent pathway without inducing excessive NF-κB-mediated inflammatory responses, thereby contributing to mucosal homeostasis [91]. Given the important role of STING in immune surveillance and host defense, the therapeutic efficacy of FMT may also depend on the host STING activation status and the successful engraftment of exogenous microbiota. One study demonstrated that FMT achieved optimal therapeutic efficacy in mice with pre-existing STING hyperactivation. Although increased STING expression initially correlated with a higher percentage of mucosal macrophages, STING activation paradoxically promoted a significant reduction in the proportions of pro-inflammatory Th1 cells, Th17 cells, and M1/M2 macrophage ratio following FMT treatment. The shift was also accompanied by enhanced colonization of *Lactobacillus*, which facilitated the restoration of intestinal homeostasis. These findings further suggest that patients with severe UC characterized by high baseline STING activation may represent a preferential responder population for FMT clinical therapy [81]. Collectively, these findings indicate that the biological consequences of STING activation in response to gut microbiota are not unidirectional, but rather are highly context-dependent and influenced by multiple factors, including the intensity and duration of STING signaling, host genetic susceptibility, as well as the composition and metabolic activity of distinct microbial communities.

Loss of the *cGAS* or *STING* may alter the composition of the gut microbiota, potentially through its role in reshaping the intestinal immune microenvironment. One study reported that *cGAS*-deficient mice exhibited attenuated colonic inflammation accompanied by increased diversity of beneficial bacterial taxa [60]. In contrast, STING signaling facilitated the engraftment and expansion of beneficial *Lactobacillus* species and their associated microbial consortia in the gut, leading to improved intestinal inflammatory outcomes [81]. In *STING^−/−^* mice, the abundance of the pathogenic genus *Desulfovibrio* (Proteobacteria phylum) was significantly increased, whereas the abundances of beneficial genera, including *Bifidobacterium* (Actinobacteria phylum) and *Allobaculum* (Firmicutes phylum), were markedly reduced [80]. This dysbiosis was accompanied by a reduction in short-chain fatty acid (SCFA)-producing bacteria, resulting in a deficiency of luminal acetate and butyrate [92]. Both acetate and butyrate exert anti-inflammatory effects in the intestine. Acetate promotes intestinal IgA production [92], while butyrate can suppress STING-activation-associated endoplasmic reticulum (ER) stress in intestinal epithelial cells, thereby decreasing the infiltration of pathogenic CD4^+^ tissue-resident memory T (TRM) cells and alleviating colitis [93]. Furthermore, tryptophan metabolites acting as aryl hydrocarbon receptor (AHR) ligands induce the nuclear translocation of STING to activate the AHR transcription factor. This nuclear signaling stimulates interleukin-17 (IL-17) and interleukin-22 (IL-22) secretion by type 3 innate lymphoid cells (ILC3s) and Th17 cells, thereby maintaining microbial homeostasis. Notably, the nuclear function of STING in activating AHR is not merely an alternative pathway but is subject to competitive inhibition by the classical cytoplasmic cGAS/STING signaling axis [94] (Figure 2).

## 5. Pharmacological Targeting of the cGAS/STING Pathway in Ulcerative Colitis

### 5.1. Synthetic Inhibitors

Small-molecule inhibitors targeting the cGAS/STING pathway represent the most direct strategy for therapeutic intervention in UC. As summarized in Table 1, current synthetic inhibitors can be broadly classified into direct cGAS inhibitors, direct STING inhibitors, and indirect pathway modulators targeting upstream DNA sensing or downstream signaling components. However, efforts to achieve optimal therapeutic efficacy face substantial translational bottlenecks, largely owing to species-dependent target variations, low gastrointestinal bioavailability, and the systemic toxicity associated with indiscriminate pathway blockade. Recent structural optimizations and advanced tissue-targeted delivery strategies have been explored to circumvent these limitations.

Among the currently available small-molecule inhibitors targeting cGAS, RU.521 is the most extensively characterized candidate. It occupies the catalytic pocket of cGAS and reduces its affinity for ATP, thereby exerting a highly selective inhibitory effect. However, frequent, high-dose intraperitoneal administration severely limits its translational potential, and its low bioavailability and susceptibility to degradation in the gastrointestinal tract pose major challenges for oral treatment of IBD. To overcome these obstacles, nanotechnology-based drug delivery systems have been developed in recent years. In one study, RU.521 was incorporated into programmable micelles assembled from hyaluronic acid–stearic acid conjugates containing ROS-cleavable thioketal linkages. The micelles enhanced drug delivery to inflamed intestinal tissue and suppressed cGAS–STING signaling in acute DSS-induced colitis [67]. A subsequent study evaluated orally administered, inflammation-triggered RU.521 micelles in gnotobiotic altered Schaedler flora IL-10-deficient mice colonized with either *Escherichia coli* 1D or *Helicobacter bilis*. Micelle monotherapy improved disease in the moderately inflamed *E. coli* 1D model, whereas the more severe *H. bilis*-associated disease required combination treatment with an anti-IL-12p40 antibody to achieve a comparable therapeutic response. These findings suggest that local cGAS inhibition may be sufficient in moderately active intestinal inflammation but may require combination therapy when redundant inflammatory circuits are strongly activated. Unlike RU.521, compound 3 covalently binds to Cys419 of mouse cGAS. When administered intraperitoneally at 20 mg/kg once daily during DSS exposure, it reduced clinical and histological manifestations of acute colitis [95]. However, Cys419 is not conserved in human cGAS, leading to weaker activity against human cGAS. Thus, its limited applicability to humans highlights the need to develop human-specific cGAS inhibitors. G108, G140, and G150 selectively inhibit human cGAS, suppressing 75–95% of cGAS-dependent responses in primary human macrophages with limited effects on cGAMP–STING or RIG-I signaling [96]. CU-32 and CU-76 also selectively inhibit DNA-induced cGAS signaling in THP-1 cells, but their binding mechanisms remain unclear. Moreover, neither compound has undergone in vivo pharmacokinetic or efficacy evaluation in experimental colitis [97].

Among synthetic small-molecule STING inhibitors, covalent inhibitors targeting Cys91 are the most common. H-151, which is active in both mice and humans, covalently binds to cysteine 91 (Cys91) of STING, thereby blocking its palmitoylation and subsequent activation and suppressing downstream signaling. Notably, H-151 was shown to be more potent than SN-011 and C-176 in J774 cells. When encapsulated in oral lipid nanocapsules, it significantly alleviated DSS-induced colitis, reduced TBK1 activation and the expression of inflammatory mediators, including TNF-α and CXCL10, and promoted mucosal healing [98]. GHN105, another orally bioavailable covalent STING inhibitor that also targets Cys91, dose-dependently inhibited the cGAS/STING pathway and type I interferon responses, and reversed pathological features of acute colitis. Importantly, the study also included delayed-treatment experiments and demonstrated in situ engagement of STING in diseased tissue, providing stronger pharmacodynamic evidence. GHN105 therefore represents one of the more advanced preclinical STING inhibitors. Nevertheless, its relatively high oral dose and validation primarily in acute DSS injury remain important limitations [99]. Palbociclib illustrates a drug-repurposing approach to direct STING inhibition. In addition to its established CDK4/6 activity, palbociclib directly bound STING near Tyr167 and interfered with STING homodimerization, cyclic dinucleotide binding, and intracellular trafficking [100]. It ameliorated DSS-induced colitis, and genetic experiments showed that the anti-inflammatory effect was dependent, at least in substantial part, on STING. However, palbociclib is not a STING-selective drug. Its canonical effects on CDK4/6, cell-cycle progression, epithelial proliferation, and immune-cell expansion may contribute to both therapeutic and adverse effects during long-term treatment. Therefore, its utility in UC will depend on whether STING inhibition can be achieved at exposures that do not substantially impair epithelial renewal or hematopoietic function [100]. Collectively, these diverse inhibitory mechanisms provide multiple strategies for targeting the STING pathway in the treatment of UC.

In addition to the direct cGAS or STING inhibitors described above, synthetic small molecules that indirectly block signal transduction by targeting upstream epigenetic regulators or downstream key kinases have also shown therapeutic potential. Azidothymidine (AZT) inhibited the reverse transcription of endogenous retroelements, reducing the production of cytosolic cDNA (a ligand for cGAS) and consequently suppressing the cGAS/STING/NF-κB pathway and M1 macrophage polarization, ultimately alleviating DSS-induced colitis [101]. Panobinostat, a histone deacetylase (HDAC) inhibitor, reduced the protein expression levels of cGAS, STING, and IRF3 and suppresses their phosphorylation in THP-1 cells, thereby alleviating DSS-induced colitis [102]. However, both AZT and panobinostat may affect biological processes beyond the cGAS pathway because of their broad effect. Regarding TBK1/IKKε inhibitors, BX795 suppressed DSS-induced colitic symptoms in vivo at a dose as low as 1 mg/kg [103]. However, it also affects AKT, JAK2/STAT1, AP-1 and IRF3 signaling. In contrast, another TBK1/IKKε inhibitor, Amlexanox (ALX), at 50 mg/kg daily, aggravated DSS colitis and promoted Th17 cell differentiation, despite its concurrent ability to inhibit dendritic cell (DC) generation and M1 macrophage polarization [104] (Table 1). The comparison between these two drugs therefore does not establish a simple pro-inflammatory or anti-inflammatory role of TBK1 in colitis. Given the differences in target selectivity and additional molecular activities of these compounds, variations in dose, treatment timing, and cellular context may further contribute to the distinct outcomes observed.

Overall, current evidence is predominantly limited to preclinical, acute DSS-induced colitis models with short-term protocols. It remains fundamentally unclear whether sustained pharmacological inhibition of the cGAS/STING pathway can maintain long-term therapeutic efficacy without compromising its physiological functions in DNA surveillance and mucosal immune defense. Therefore, future drug development must prioritize human-specific target validation, strict target-engagement analyses, and the optimization of intestine-targeted delivery strategies to balance safety with therapeutic outcomes.

**Table 1:** Summary of synthetic small-molecule inhibitors that regulate the cGAS/STING signaling in UC.

Target	Inhibitors	Molecular Mechanism	Effective Dose (In Vivo)	Experimental Models	Associated Phenotype	Reference
cGAS	Ru.521	Inhibits the catalytic activity of ATP and GTP sites on cGAS	100 μM	DSS-induced acute colitis	ROS, MPO ↓; IL-1β, TNF-α, IL-6 ↓; FITC ↑	[68]
cGAS	Compound 3	Covalently binds to Cys419 of cGAS	20 mg/kg	DSS-induced acute colitis	IL-1β, TNF-α, IL-6 ↓	[95]
STING	H-151	Covalently binds Cys91 of STING, inhibits palmitoylation	10.4 mg/kg(IP) or 24 mg/kg(OR)	DSS-induced acute colitis	TBK1, p-TBK1 ↓, p-STING ↓; TNF-α, CXCL10, IL-1β, MIP-1α ↓; MPO ↓	[98]
STING	GHN105	Selectively engages the membrane-proximal Cys91 residue of STING	100 mg/kg	DSS-induced acute colitis, Thp-1	MPO, TNF-α ↓	[99]
STING	Palbociclib	Targets Y167 of STING to block its dimerization, its binding with cyclic dinucleotides, and its trafficking	10 mg/kg	DSS-induced acute colitis, Peritoneal macrophages	IL-6, IL-1β, IFN-β ↓	[100]
TBK1/IKKε	BX795	Inhibits catalytic activity of TBK1/IKKε	1 mg/kg	DSS-induced acute colitis, Raw264.7	p-TBK1, p-STAT1 ↓; IL-1β, TNF-α ↓	[103]
TBK1/IKKε	Amlexanox (ALX)	Selective inhibitor of TBK1 and IKKε	50 mg/kg	DSS-induced acute colitis	TBK1, IRF3, P65 ↓; IL-10, TGF-β ↓; IL-1β, IFN-α ↑; sIgA ↓; Th17 ↑; M1 macrophage ↓	[104]
cDNA	Azidothymidine (AZT)	Limits retroelement cDNA synthesis and suppresses cGAS/STING activation	5 mg/kg	DSS-induced acute colitis, Raw264.7	Mervl, Line-1 ↓; p-STING/STING, p-TBK1/TBK1, p-IRF3/IRF3, p-P65/P65 ↓; M1 macrophage ↓	[101]
HDAC	Panobinostat	Reduces cGAS and STING protein expression via HDAC inhibition	5 mg/kg	DSS-induced acute colitis	HDAC3, cGAS, STING ↓; TNF-α, CXCL10, IL-1β, IL-6 ↓	[102]

MPO, myeloperoxidase; FITC, fluorescein isothiocyanate; CXCL10, C-X-C motif chemokine ligand 10; HDAC, histone deacetylase; HDAC3, histone deacetylase 3; IKKε, inhibitor of nuclear factor kappa-B kinase subunit epsilon; IP, intraperitoneal; OR, oral administration; MIP-1α, macrophage inflammatory protein-1 alpha; sIgA, secretory immunoglobulin A; Th17, T helper 17 cells; cDNA, complementary DNA; Line-1, long interspersed nuclear element-1; Mervl, murine endogenous retrovirus-like.

### 5.2. Natural Products

Natural products have emerged as important candidates for modulating the cGAS/STING pathway due to their chemical diversity and pleiotropic biological activities. As summarized in Table 2, natural products regulate this pathway through several distinct mechanisms, including direct inhibition of cGAS/STING activation, regulation of downstream signaling molecules, promotion of DNA clearance, and modulation of microbiota-derived metabolites.

Several natural compounds directly target key signaling proteins or interfere with critical signal transduction steps, thereby blocking pro-inflammatory cascades. For example, toosendanin directly binds to STING in macrophages, inhibits its oligomerization, and also interacts with LYN kinase, thereby suppressing downstream AKT-dependent signaling, a pathway in which AKT phosphorylation is critically required for STING activation. In vivo experiments further demonstrated that toosendanin inhibited STING pathway activation and reduced macrophage infiltration in DSS-induced colitis mice [105]. However, this dual-target activity makes it difficult to determine how much of the in vivo effect results from direct STING inhibition rather than LYN-dependent modulation of macrophage activation. Kurarinone, a flavonoid isolated from Sophora flavescens, interferes with the physical interaction between STING and IRF3 without affecting STING oligomerization, thereby reducing the release of pro-inflammatory cytokines and interferons, an effect that was validated in vivo [106]. Therefore, it should be described as a STING-targeting natural product that disrupts STING–IRF3 signal transmission rather than as a site-defined STING inhibitor. Licochalcone D, a compound isolated from licorice, was identified as a STING inhibitor that covalently modifies Cys148 of STING, suppresses STING oligomerization, blocks TBK1 recruitment and nuclear translocation of IRF3/NF-κB. It suppressed inflammatory pathology not only in DSS-induced colitis but also in TREX1-deficient inflammatory disease and azoxymethane/DSS-induced colitis-associated colorectal cancer [107]. However, covalent modification raises selectivity concerns and requires further evaluation of its therapeutic window. Quercetin reduced disease activity, epithelial permeability, and histological injury by decreasing cGAS/STING expression and TBK1/IRF3 phosphorylation. It also shifted macrophage polarization away from an M1-dominant phenotype, and restored the expression of barrier proteins such as ZO-1 and occludin [108]. However, direct binding to cGAS or STING was not demonstrated. Decursin, the main active ingredient of Angelica gigas Nakai, alleviated DSS-induced colitis in mice by inhibiting cGAS-STING pathway activation, restoring the expression of tight junction proteins and reducing serum pro-inflammatory cytokines [109]. Trans-ferulic acid was encapsulated in pectin-based nanomaterials to further enhance the colon-targeting efficiency of natural products via nanodelivery technology and this formulation effectively alleviated UC, particularly by inhibiting cGAS/STING activation [110].

Certain natural products indirectly modulate this pathway by acting as upstream transcriptional regulators, altering protein stability or simultaneously targeting multiple downstream signaling nodes. For example, curcumol, acting as a PXR ligand, downregulates the STING pathway through nuclear receptor-mediated transcriptional regulation. Genetic loss of PXR markedly reduced or abolished the protective effect of curcumol, while chromatin-immunoprecipitation experiments supported PXR occupancy at the STING regulatory region [111]. Therefore, it acts through a PXR–STING transcriptional axis. Physcion appears to connect mitochondrial regulation, innate DNA sensing, and inflammatory cell death. It increased expression or activity of the mitochondrial deacetylase SIRT3 and concomitantly reduced cGAS–STING activation and intestinal epithelial PANoptosis. Loss-of-function experiments supported a requirement for SIRT3 in the protective response, suggesting that physcion acts through a SIRT3-dependent mechanism upstream of, or at the level of, cGAS regulation [112]. In addition to single compounds, traditional Chinese medicine formulas also offer a synergistic approach by targeting multiple downstream nodes of this cascade. Sini San ameliorated chronic experimental colitis in mice by selectively suppressing type I interferon responses; further studies revealed that Sini San inhibited IFN-stimulated gene expression through modulation of TBK1 and IRF3 dependent signaling as well as direct inhibition of STAT1 and STAT2 activation [113]. Wumei Wan inhibited STING/IRF3 pathway, alleviated intestinal inflammation and restored intestinal stem-cell-associated regenerative responses [114]. However, the current evidence does not establish that inhibition of STING is necessary or sufficient for the effect of the formula.

Another important strategy by which natural products downregulate the cGAS/STING pathway is the elimination of endogenous DAMPs. For instance, Brucea javanica oil emulsion (BJOE), a botanical anti-inflammatory agent, stabilized deoxyribonuclease 2 (DNase2) to clear inflammatory DNA triggers, thereby inhibiting the cGAS/STING pathway and restoring intestinal epithelial barrier integrity [115]. Nevertheless, BJOE is a multicomponent preparation, and the constituent responsible for DNase2 regulation has not been fully identified. Another study using pectin to enhance the therapeutic potential of caffeic acid, a rectal gel was developed that reduced mtDNA release, modulated the cGAS/STING pathway, and decreased oxidative stress and apoptosis [116].

Some natural products indirectly modulate the cGAS/STING axis by remodeling the gut microbiota. For example, lily polysaccharides restored the gut microbiota and increased elevate N8-acetyl spermidine (N8AS) levels in mice with DSS-induced colitis and exogenous N8AS supplementation suppressed the activation of cGAS, STING, TBK1, IRF3 and alleviated colitis [117]. Ophiopogon japonicus polysaccharides (OJP) increased *Lactobacillus salivarius*, which promoted the production of chenodeoxycholic acid (CDCA). CDCA may alleviate colitis by binding to STING and inhibiting the NF-κB pathway [118]. However, direct CDCA-STING binding requires confirmation using orthogonal biophysical methods. Bile acids act through multiple pathways, so specific controls are needed to confirm STING specificity. Pueraria flavonoids, the main active components of Pueraria lobata, reduced cGAS/STING activation and improved intestinal barrier function, presumably through modulation of the gut microbiota and its metabolites [119].

Overall, natural products regulate the cGAS/STING pathway through heterogeneous mechanisms, but direct target engagement has been established for only a limited number of compounds. For many agents, pathway suppression may be secondary to broader antioxidant, barrier-protective, or microbiota-modulating effects. Further development therefore requires identification of active constituents, mechanistic validation, chemical standardization, and characterization of intestinal exposure. Although their pleiotropic effects complicate target attribution, they may also be advantageous in the multifactorial setting of UC.

## 6. Conclusions and Perspective

Despite significant therapeutic advancements, the chronic and relapsing nature of UC continues to present a formidable clinical challenge, largely due to the incomplete resolution of underlying mucosal inflammation. Aberrant activation of the cGAS/STING signaling pathway has emerged as a crucial driver in the pathogenesis of various gastrointestinal disorders. However, this pathway functions as a biological double-edged sword, orchestrating pathological inflammatory cascades while simultaneously maintaining epithelial homeostasis and microbial stability. This review systematically discusses the current knowledge of the dual role of the cGAS/STING pathway in UC pathogenesis and summarizes recent progress in the development of drugs targeting this pathway.

Based on the current analytical framework, we propose several critical directions for future research. Firstly, the functional spatiotemporal heterogeneity of cGAS/STING across distinct intestinal cell types (e.g., epithelial cells, macrophages, dendritic cells, and T cells) must be systematically investigated. It is imperative to clarify the distinction between baseline physiological signaling and pathological overactivation, thereby defining the biological outcomes associated with different activation intensities. Secondly, because this pathway is readily activated by bacterial DNA, it is essential for researchers to optimize animal models and develop mouse models that more closely recapitulate the human gut microbiota to enhance the reliability and generalizability of experimental findings [120,121]. Critically, confounding variables originating from environmental and microbial sources must be rigorously controlled. Thirdly, the exploration of intervention strategies targeting specific signaling nodes warrants detailed investigation. Comparing different intervention modalities (e.g., competitive inhibitors, covalent modifiers, and disruptors of protein–protein interactions) will help identify optimal therapeutic windows and strategies. Notably, the multiple post-translational modifications of cGAS and STING represent promising targets for fine-tuned regulation. Finally, beyond their well-characterized signaling partnership, the non-canonical functions of cGAS and STING that operate independently require further elucidation, particularly regarding how these activities intersect with classical inflammatory cascades in UC.

From a translational perspective, species-dependent structural and functional differences in cGAS and STING limit the direct extrapolation of findings from murine colitis models to human UC. Furthermore, while most animal studies to date have been limited to short-term interventions, UC is a chronic disease requiring long-term disease control. Because cGAS/STING signaling contributes to host defense against enteric pathogens, maintenance of microbial homeostasis, and tumor immunosurveillance, a systematic assessment of the long-term safety of cGAS/STING-targeted drugs is urgently needed to evaluate the risks of infection, persistent microbiota disturbance, and intestinal tumorigenesis. Thus, a considerable translational gap remains before existing findings can safely enter clinical practice. Ultimately, a more thorough and context-aware investigation of the cGAS/STING pathway will not only deepen our fundamental insight into mucosal immunity but also will also be essential for developing precise and safe therapeutic strategies.

## Figures and Tables

**Table 2:** Summary of Natural products that regulate the cGAS/STING signaling in UC.

Agent/Formulation	Experimental Models	Effective Dose	Associated Phenotype	References
Toosendanin	DSS-induced acute colitis mice, THP-1, RAW264.7, BMDM	0.5 mg/kg	p-STING, p-TBK1, p-IRF3, p-P65 ↓; IFNB1, CXCL10, IL-6, TNF-α ↓; STING oligomerization ↓	[105]
Kurarinone	DSS-induced acute colitis mice, BMDMs and THP-1 cells	10 mg/kg	p-STING, p-TBK1, p-IRF3 ↓; IFNβ, IL-1β, CXCL10, IL-6, TNF-α, ISG15 ↓; STING-IRF3 interaction ↓	[106]
Licochalcone D	DSS-induced acute colitis mice, THP-1, RAW264.7 cells	20 mg/kg	STING Cys148 covalent modification ↑, STING oligomerization ↓; p-STING, p-TBK1, p-IRF3, p-NF-κB, p-STAT3 ↓	[107]
Quercetin	DSS-induced acute colitis mice, RAW264.7 cells and BMDMs	30 mg/kg	cGAS, STING, p-TBK1, p-IRF3 ↓; M1 macrophages ↓, M2 macophages ↑; CXCL10, TNF-α ↓; IL10, CCL17 ↑; ZO1, Occludin ↑	[108]
Decursin	DSS-induced acute colitis mice	10 mg/kg	cGAS, STING, p-STING, p-TBK1, NF-κB p65 ↓; TNF-α, IL-1β, IL-6 ↓; ZO-1, Occludin, claudin 1 ↑	[109]
Ferulic acid	DSS-induced acute colitis mice	50 mg/kg	γH2AX, cGAS, STING, p-STING ↓; TNF-α, IL-1β ↓; NO ↓, GSH ↑	[110]
Curcumol	DSS-induced acute colitis mice, BMDMs, LS174T, STING^−/−^ mice, DMXAA induced mice	4 g/kg	NF-κB1, TNF, IL-1β ↓; F4/80 ↓; Occludin ↓	[111]
Physcion	DSS-induced acute colitis mice, Primary IECs and HEK293T	10 mg/kg	SIRT3 ↑; cGAS, STING ↓; cGAS acetylation ↓; cGAS polyubiquitination ↑; Cleaved-Caspase-3, Cleaved-Caspase-8, GSDMD-N, p-RIPK1 ↓	[112]
Sini San	DSS-induced chronic colitis mice, RAW264.7	1.56 g/kg	IFNB1, ISG15, IFIT1, USP18 ↓; STING ↓, p-TBK1, p-IRF3, p-STAT1, p-STAT2, p-IRF7 ↓	[113]
Wumei Wan	DSS-induced acute colitis mice	8.01 g/kg	P-STING, p-IRF3, p-P65 ↓	[114]
Brucea javanica oil emulsion (BJOE)	DSS-induced acute colitis mice, NCM460	0.5 mg/kg	dsDNA ↓; cGAS, STING, p-IRF3, p-TBK1 ↓; ZO-1, Occludin, Claudin-1, E-cadherin ↑; IFN-β, TNF-α, IL-1β ↓	[115]
Caffeic acid	DSS-induced acute colitis mice, NCM460	50 mg/kg	mtDNA, cGAS, STING, IFN-γ ↓	[116]
Lily polysaccharides	DSS-induced acute mice, Raw264.7, Caco-2	150 mg/kg	gut microbial homeostasis ↑, cGAS, STING, p-IRF3, p-TBK1 ↓; IL-1β, IL-6, TNF-α ↑; Occludin, ZO-1, MUC2 ↓	[117]
Ophiopogon japonicus polysaccharides (OJP)	DSS-induced acute colitis mice	50 mg/kg	*Lactobacillus salivarius* ↑, chenodeoxycholic acid (CDCA) ↑; p-STING, p-NF-κB↓	[118]
Pueraria flavonoes	DSS-induced acute colitis mice, Caco-2	50 mg/kg	IL-1β, IL-6, TNF-α ↓; ZO-1, Occludin, Claudin-3 ↑; iNOS, COX2 ↓; mtDNA ↓; cGAS, STING, p-IRF3/IRF3 ↓	[119]

BMDMs, bone marrow-derived macrophages; CCL17, C-C motif chemokine ligand 17; COX2, cyclooxygenase-2; CXCL10, C-X-C motif chemokine ligand 10; GSH, glutathione; IFN-γ, interferon-γ; IFNB1, interferon beta 1; IFIT1, interferon-induced protein with tetratricopeptide repeats 1; iNOS, inducible nitric oxide synthase; ISG15, IFN-stimulated gene 15; MDA, malondialdehyde; NO, nitric oxide; p-STAT1/2/3, phosphorylated signal transducer and activator of transcription 1/2/3; USP18, ubiquitin-specific peptidase 18; p-RIPK1, phosphorylated receptor-interacting protein kinase 1.

## Data Availability

No new data were created or analyzed in this study. Data sharing is not applicable to this article.
